# Supplementary figures and images for: pYtags enable spatiotemporal measurements of receptor tyrosine kinase signaling in living cells
Source: eLife. 2023 May 22;12:e82863. doi: 10.7554/eLife.82863 (PMC10202457; doi:10.7554/eLife.82863)

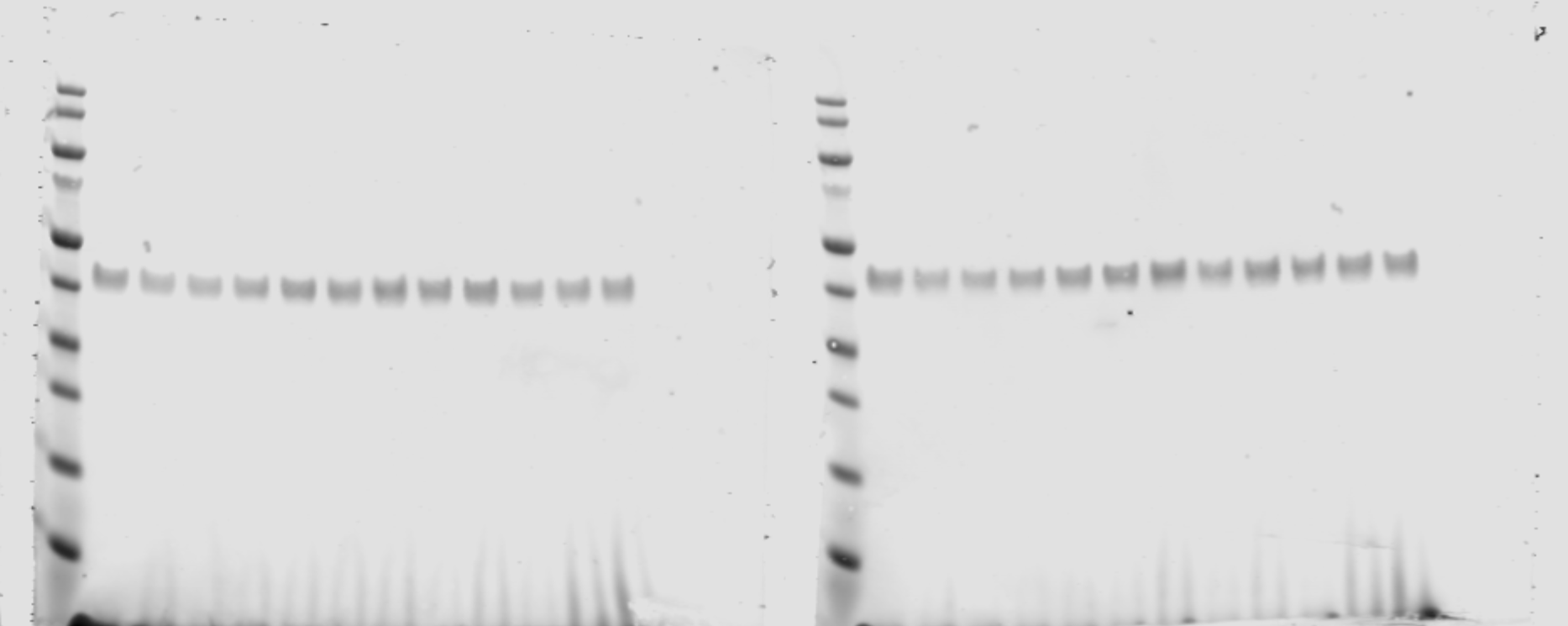

Supplement: Figure 1—source data 1. [file elife-82863-fig1-data1.zip › Figure 1 - source data 1/blot #1 actin.tif]

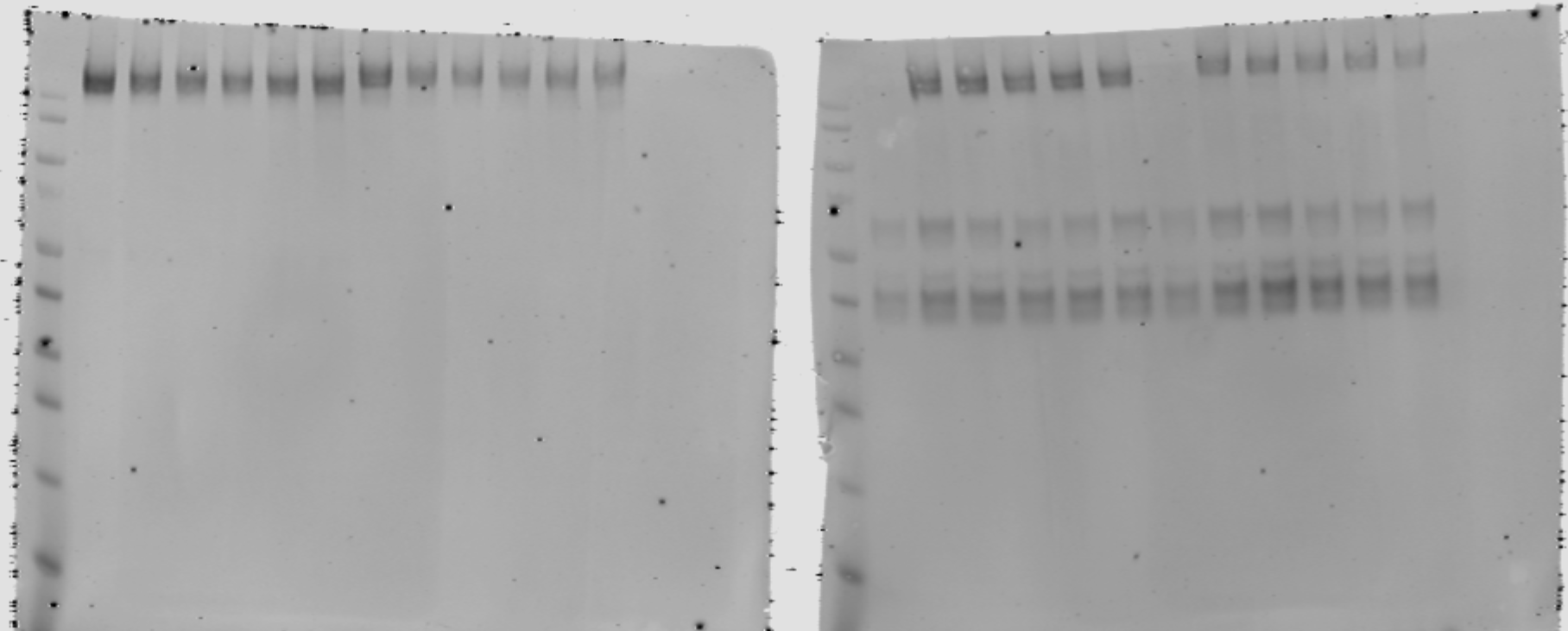

Supplement: Figure 1—source data 1. [file elife-82863-fig1-data1.zip › Figure 1 - source data 1/blot #1 EGFR.tif]

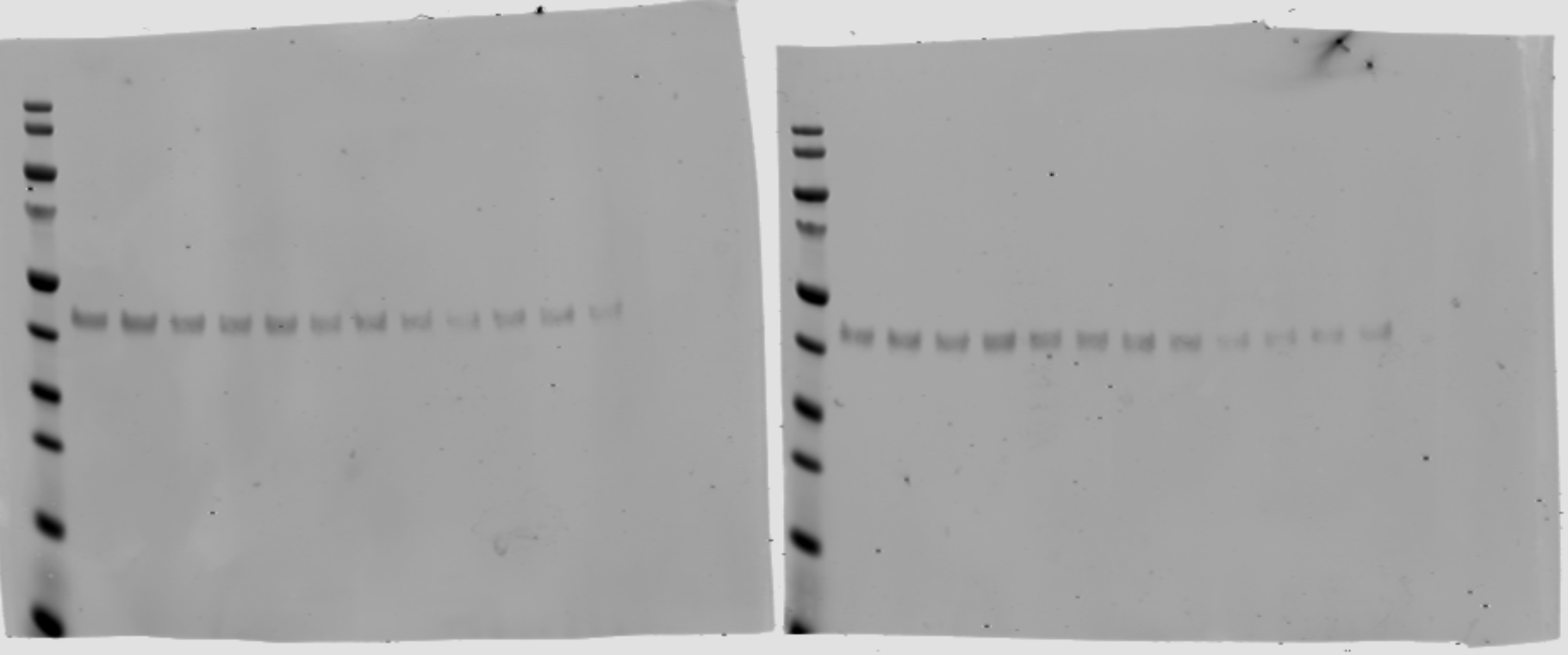

Supplement: Figure 1—source data 1. [file elife-82863-fig1-data1.zip › Figure 1 - source data 1/blot #2 actin.tif]

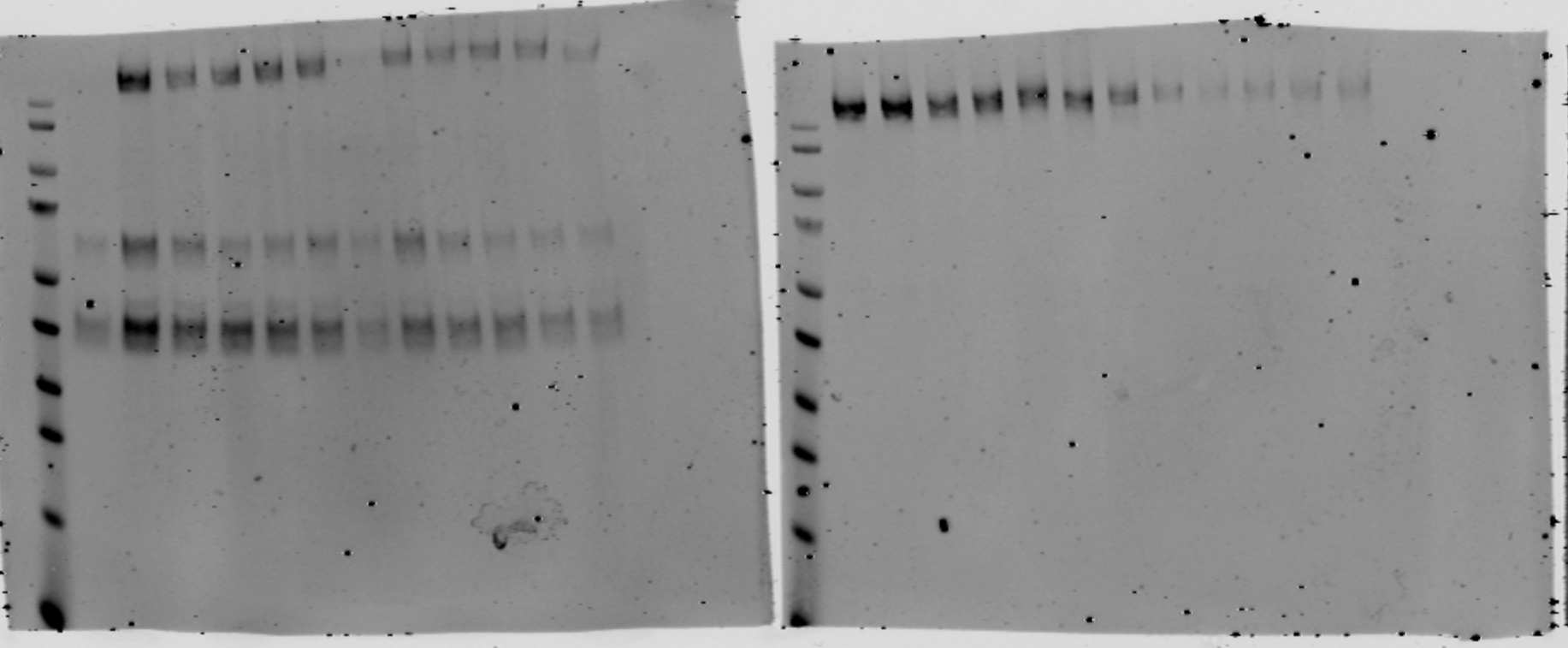

Supplement: Figure 1—source data 1. [file elife-82863-fig1-data1.zip › Figure 1 - source data 1/blot #2 EGFR.tif]

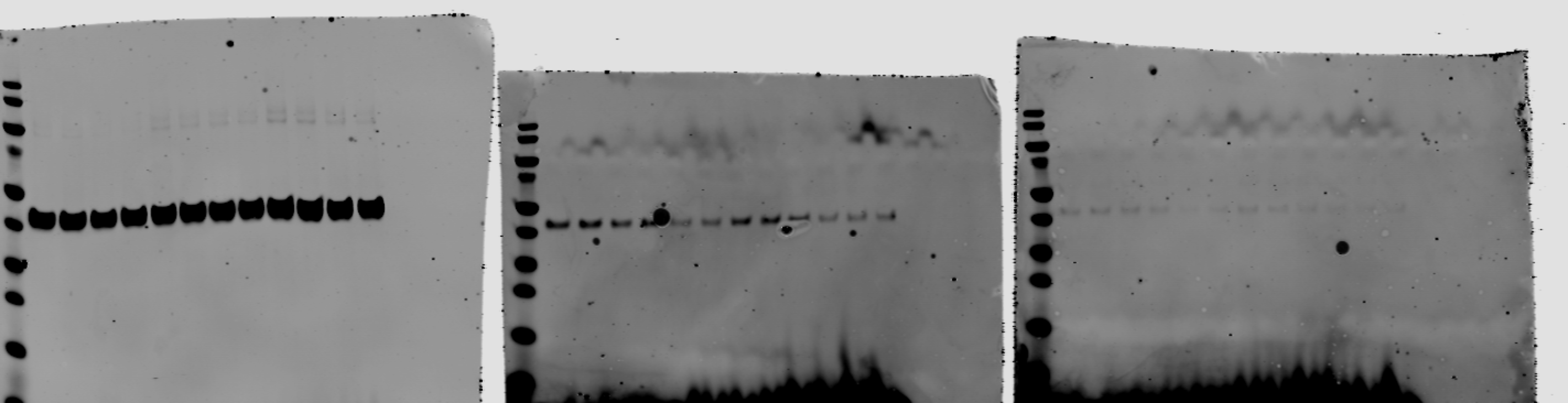

Supplement: Figure 1—source data 1. [file elife-82863-fig1-data1.zip › Figure 1 - source data 1/blot #3 actin.tif]

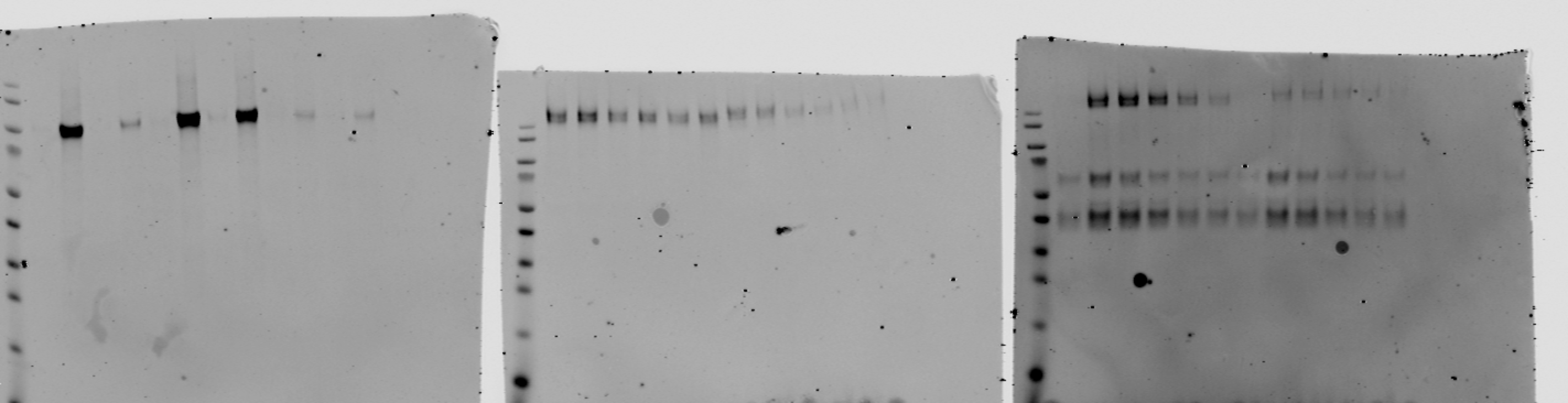

Supplement: Figure 1—source data 1. [file elife-82863-fig1-data1.zip › Figure 1 - source data 1/blot #3 EGFR.tif]

Figure 1F blot #2

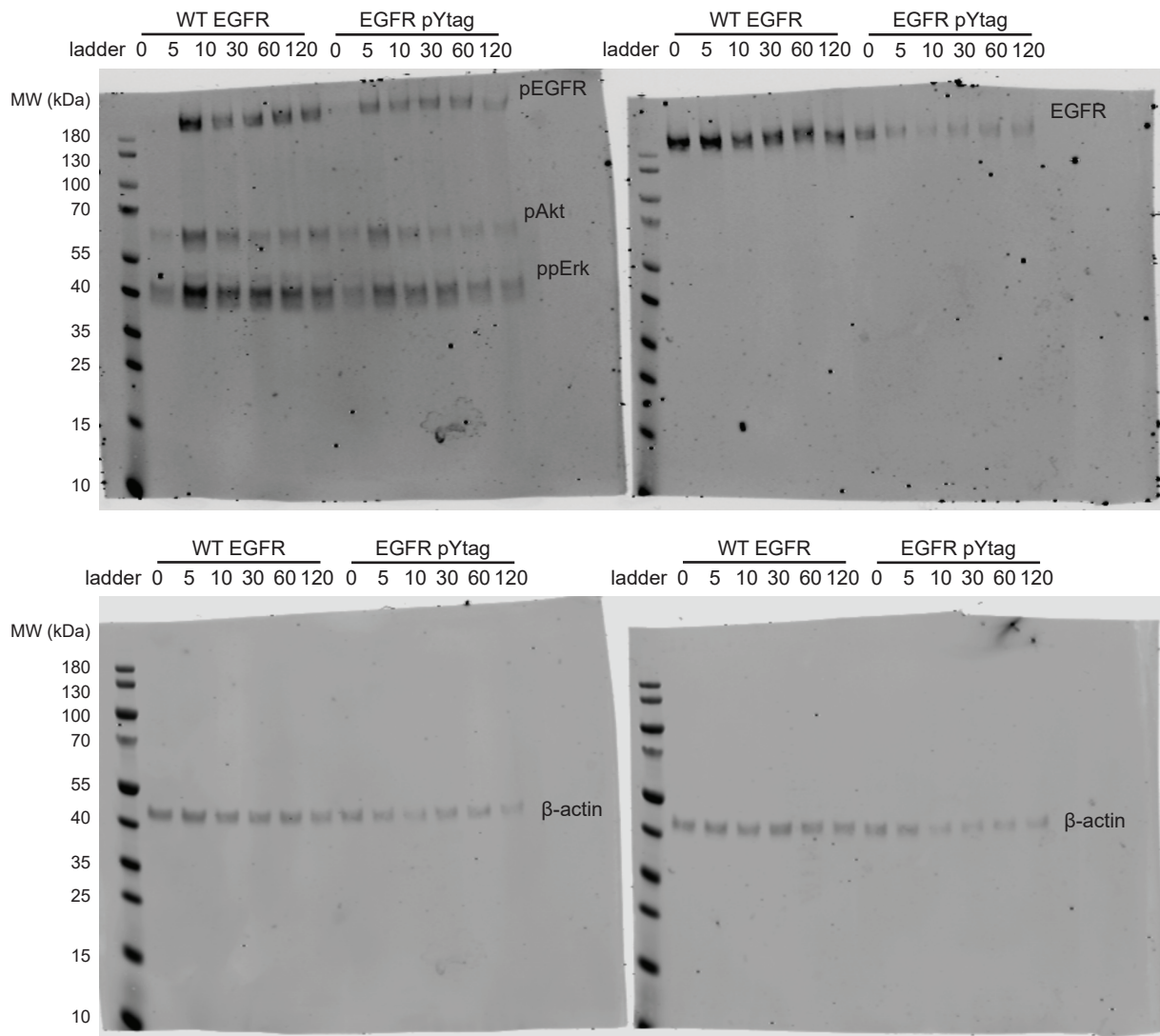

Supplement: Figure 1—source data 1. [file elife-82863-fig1-data1.zip › Figure 1 - source data 1/Figure 1 - source data 1 (blot #2).pdf]

Figure 1F blot #3

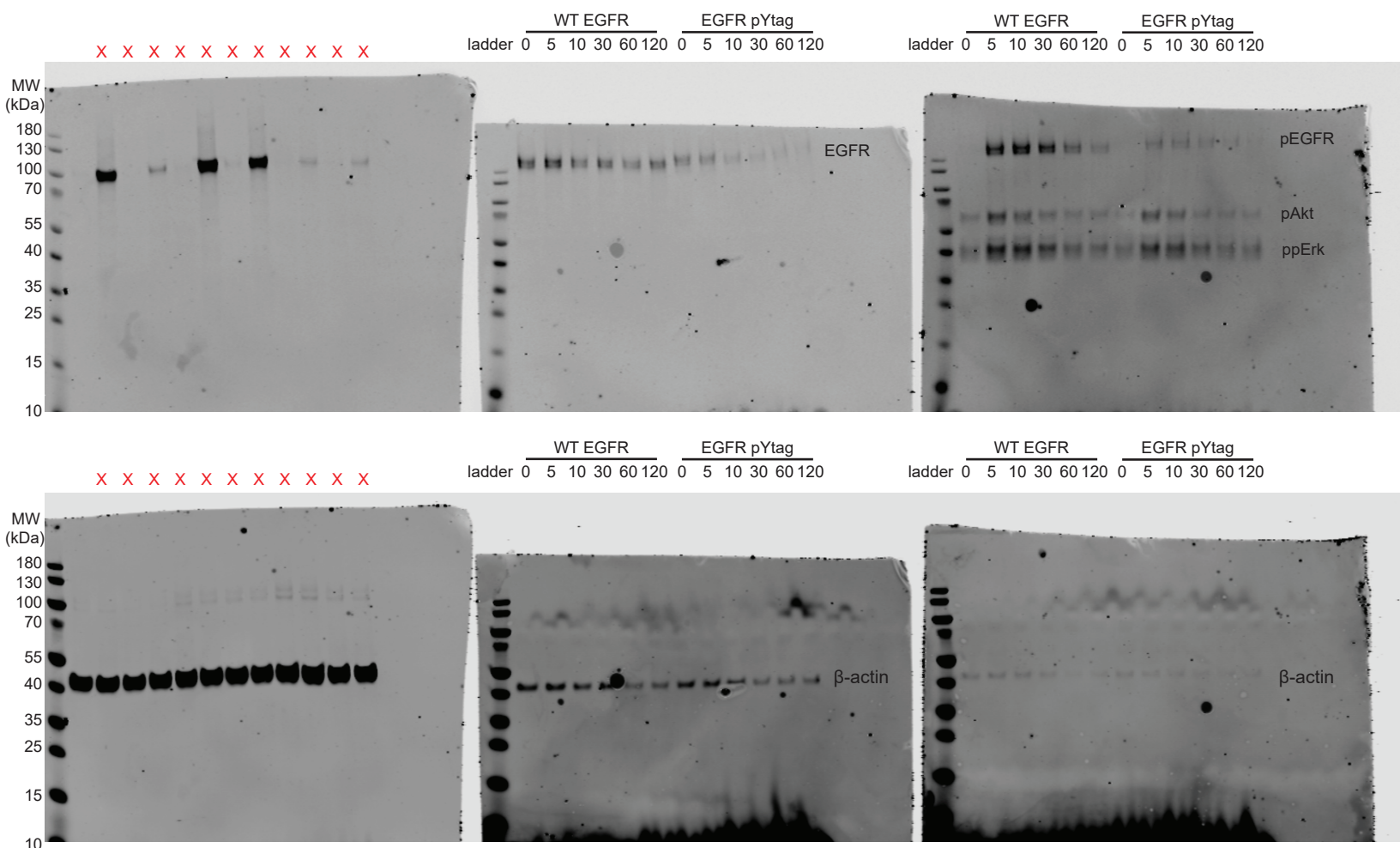

Supplement: Figure 1—source data 1. [file elife-82863-fig1-data1.zip › Figure 1 - source data 1/Figure 1 - source data 1 (blot #3).pdf]

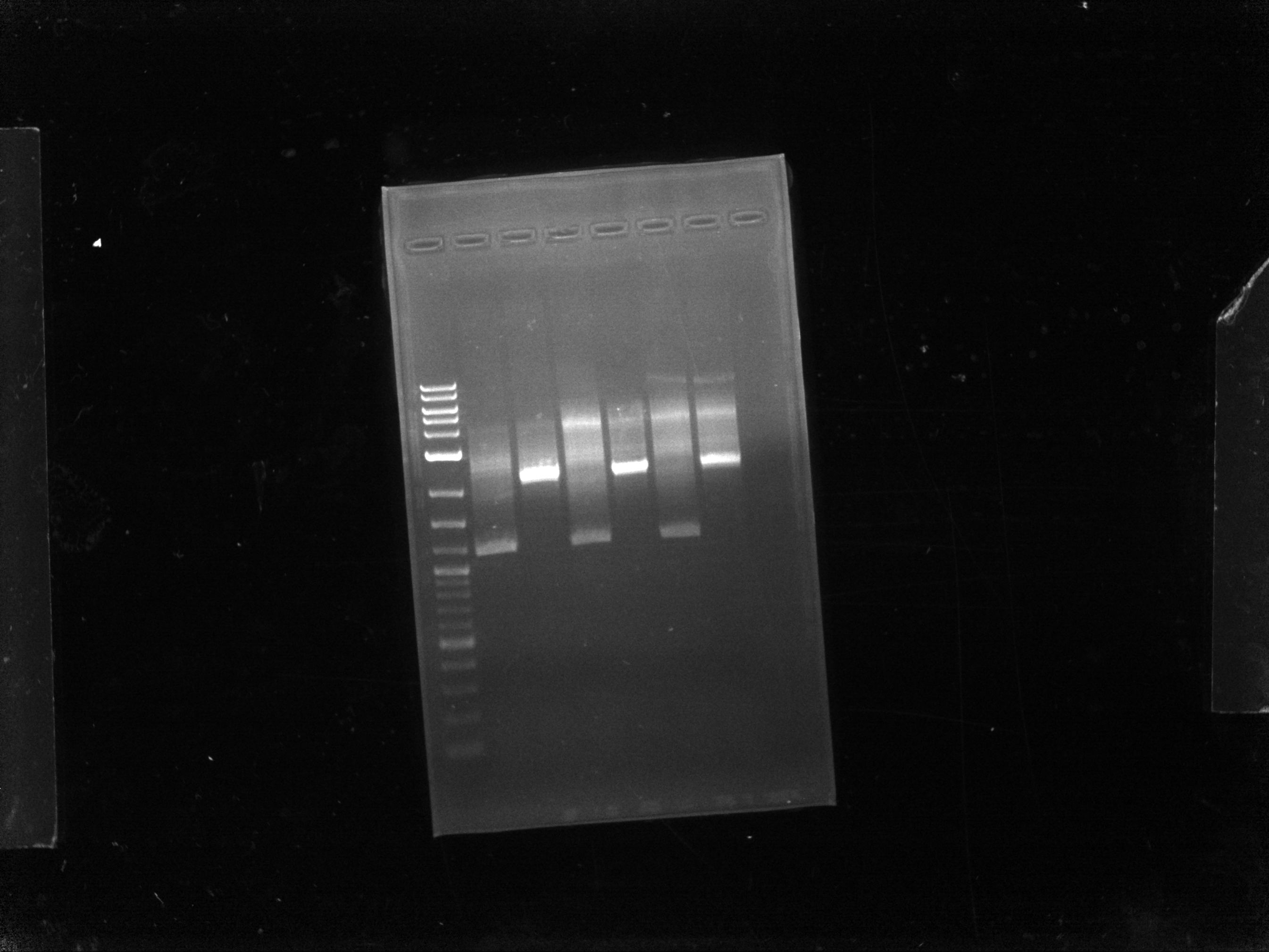

Supplement: Figure 6—source data 1. [file elife-82863-fig6-data1.zip › Figure 6 - source data 1/2022-0805-111345.tif]

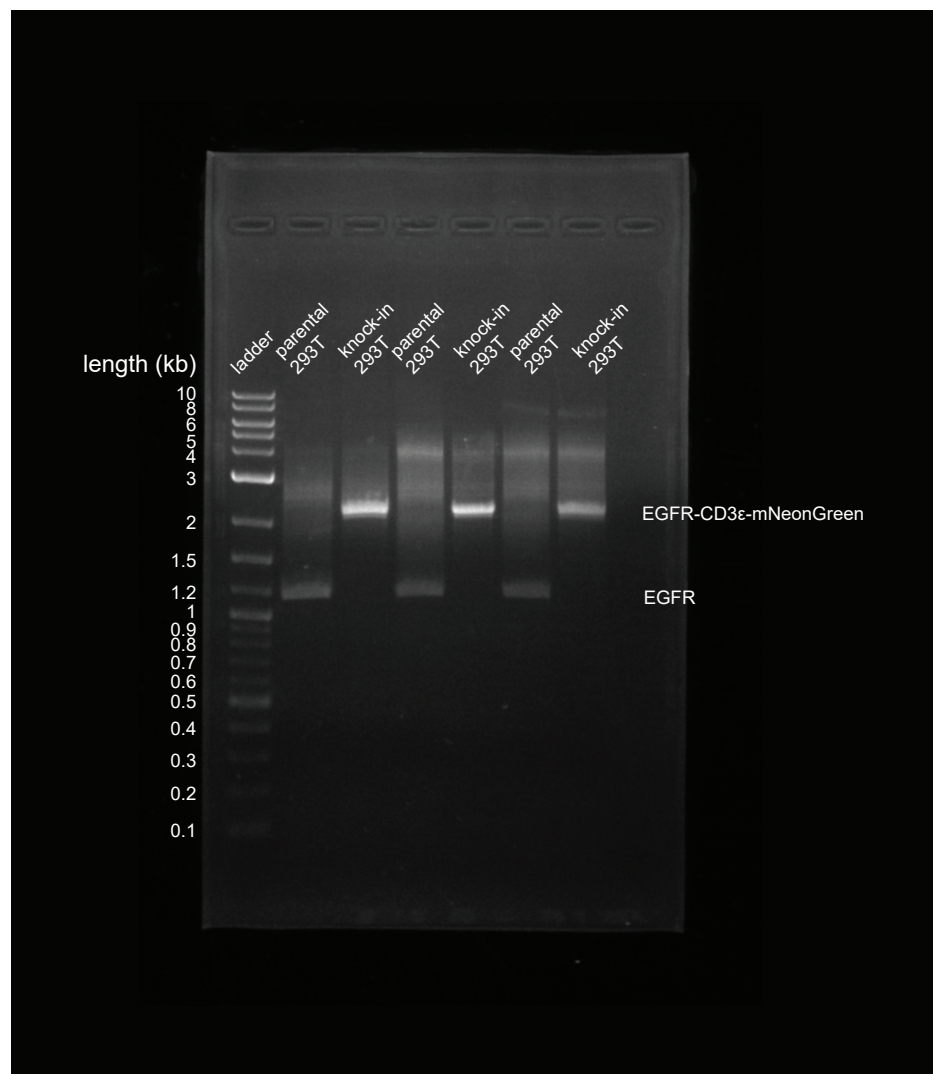

Supplement: Figure 6—source data 1. [file elife-82863-fig6-data1.zip › Figure 6 - source data 1/Figure 6 - source data 1.pdf]

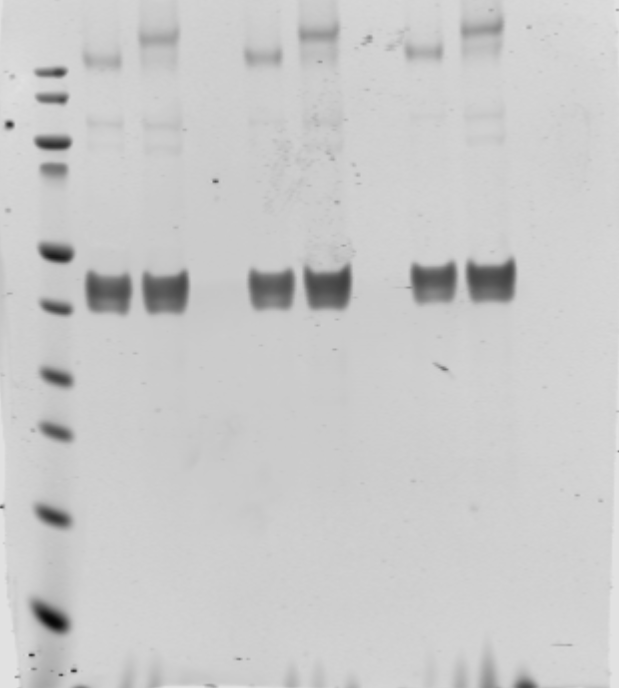

Supplement: Figure 6—source data 2. [file elife-82863-fig6-data2.zip › Figure 6 - source data 2/2022_07_23_pYtagCRISPR.tif]

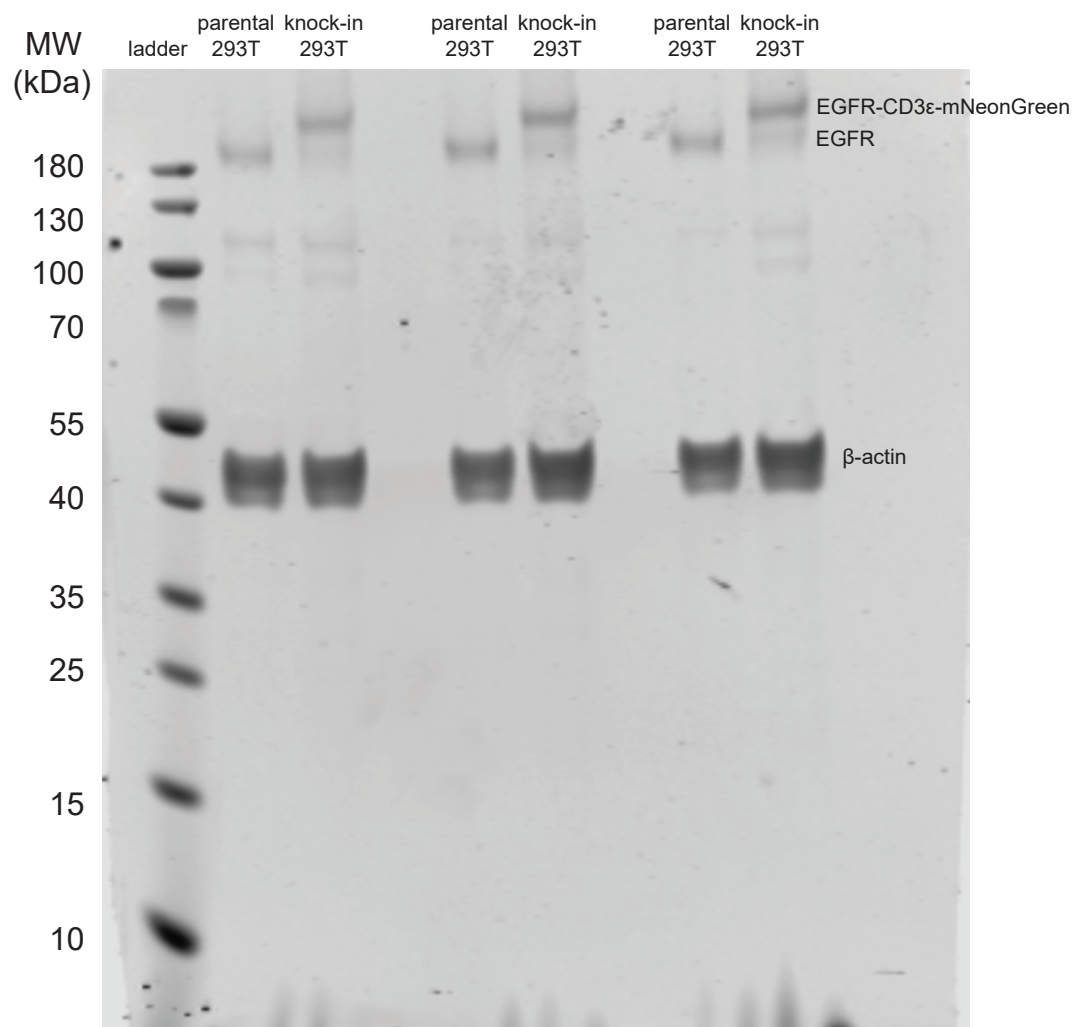

Supplement: Figure 6—source data 2. [file elife-82863-fig6-data2.zip › Figure 6 - source data 2/Figure 6 - source data 2.pdf]
