# Supplementary material for: pYtags enable spatiotemporal measurements of receptor tyrosine kinase signaling in living cells
Source: eLife. 2023 May 22;12:e82863. doi: 10.7554/eLife.82863 (PMC10202457; doi:10.7554/eLife.82863)

Figure 1F blot #1

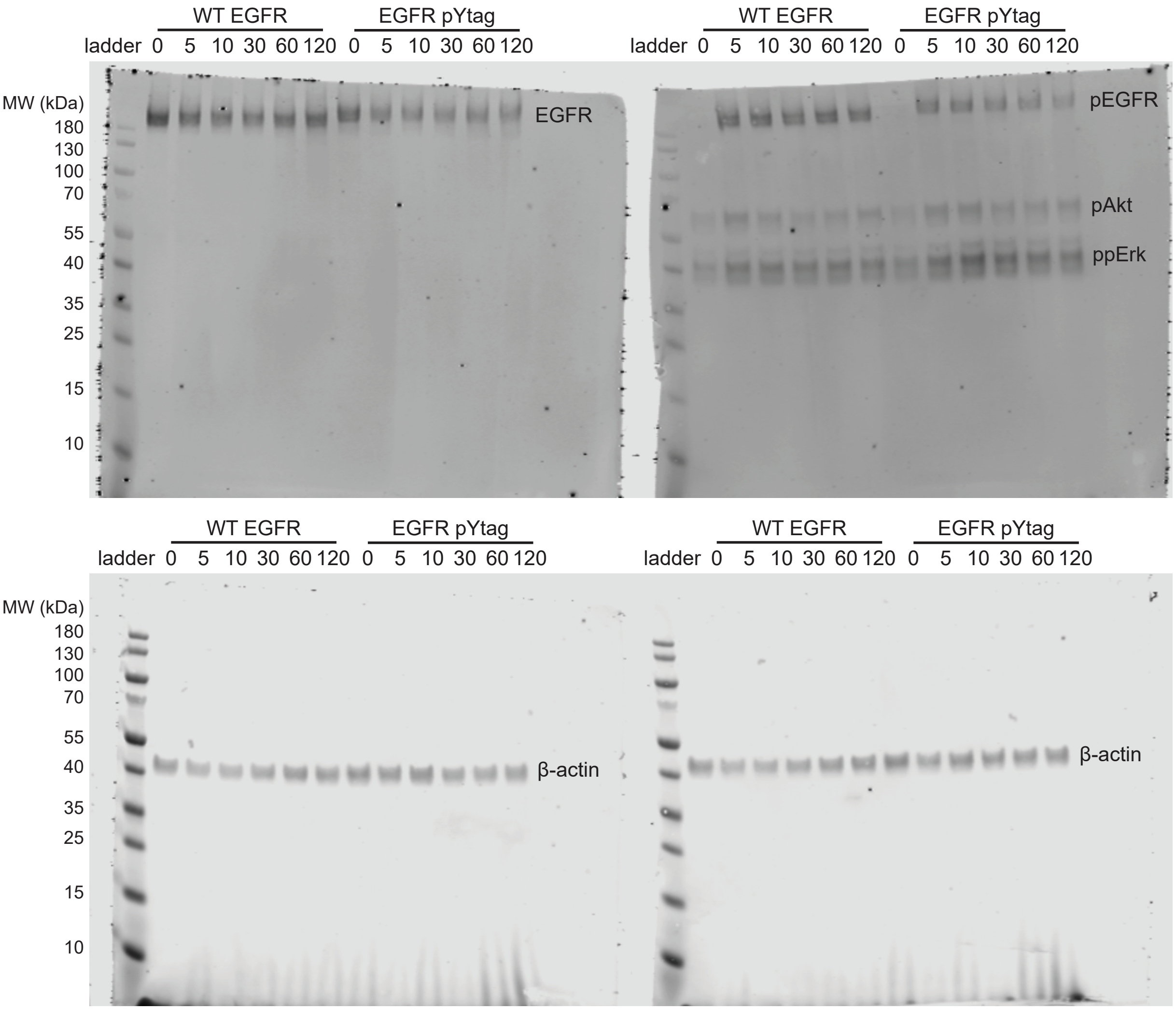

Supplement: Figure 1—source data 1. [file Figure 1 - source data 1 (blot #1).pdf › Figure 1 - source data 1/Figure 1 - source data 1 (blot #1).pdf]
